# Habitat selection of resident and non-resident gray wolves: implications for habitat connectivity

**DOI:** 10.1038/s41598-023-47815-0

**Published:** 2023-11-21

**Authors:** M. van den Bosch, K. F. Kellner, M. G. Gantchoff, B. R. Patterson, S. M. Barber-Meyer, D. E. Beyer, J. D. Erb, E. J. Isaac, D. M. MacFarland, S. A. Moore, D. C. Norton, T. R. Petroelje, J. L. Price Tack, B. J. Roell, M. Schrage, J. L. Belant

**Affiliations:** 1 Department of Fisheries and Wildlife, Michigan State University, East Lansing, MI USA; 2 Department of Biology, University of Dayton, Dayton, OH USA; 3 Ontario Ministry of Natural Resources, Wildlife Research and Development Section, Trent University, Peterborough, ON Canada; 4 Pacific Whale Foundation, Wailuku, HI USA; 5 Minnesota Department of Natural Resources, Forest Wildlife Populations and Research Group, Grand Rapids, MN USA; 6 Grand Portage Band of Lake Superior Chippewa, Biology and Environment, Grand Portage, MN USA; 7 Wisconsin Department of Natural Resources, Office of Applied Science, Rhinelander, WI USA; 8 Wildlife Division, Michigan Department of Natural Resources, Marquette, MI USA; 9 Fond du Lac Resource Management Division, Cloquet, MN USA

**Keywords:** Ecology, Animal migration, Conservation biology, Ecological modelling

## Abstract

Habitat selection studies facilitate assessing and predicting species distributions and habitat connectivity, but habitat selection can vary temporally and among individuals, which is often ignored. We used GPS telemetry data from 96 Gray wolves (*Canis lupus*) in the western Great Lakes region of the USA to assess differences in habitat selection while wolves exhibited resident (territorial) or non-resident (dispersing or floating) movements and discuss implications for habitat connectivity. We used a step-selection function (SSF) to assess habitat selection by wolves exhibiting resident or non-resident movements, and modeled circuit connectivity throughout the western Great Lakes region. Wolves selected for natural land cover and against areas with high road densities, with no differences in selection among wolves when resident, dispersing, or floating. Similar habitat selection between resident and non-resident wolves may be due to similarity in environmental conditions, when non-resident movements occur largely within established wolf range rather than near the periphery or beyond the species range. Alternatively, non-resident wolves may travel through occupied territories because higher food availability or lower human disturbance outweighs risks posed by conspecifics. Finally, an absence of differences in habitat selection between resident and non-resident wolf movements may be due to other unknown reasons. We recommend considering context-dependency when evaluating differences in movements and habitat use between resident and non-resident individuals. Our results also provide independent validation of a previous species distribution model and connectivity analysis suggesting most potential wolf habitat in the western Great Lakes region is occupied, with limited connectivity to unoccupied habitat.

## Introduction

Understanding how animals select habitat is necessary to explain and predict species distributions, facilitating population management and species conservation^1,2^. Characterizing species-habitat relationships can inform where populations can establish^3^ and identify linkages between habitat patches suitable for dispersal^4^. Whereas habitat selection and associated connectivity studies are valuable^2,4^, processes underlying habitat selection are often poorly understood^5^. Drivers of habitat selection can differ among life stages or individuals and understanding these differences can improve our understanding of habitat selection and connectivity^5,6^.

Mismatches between landscape connectivity analyses and species ecology can be mitigated by accounting for behavioral aspects that can influence movement^6,7^. Processes underlying animal movements are relevant to connectivity analyses as they influence the behavior and movements of dispersing animals^8^. Human landscape disturbances, including high human population densities and associated activities, can alter large carnivore movements^9,10^, but avoidance of human disturbance can be lower for non-resident individuals than for residents^11^. This can affect accuracy of connectivity models, as studies generally consider habitat selection across individuals as equivalent^12,13^.

Reduced avoidance of human disturbance by non-resident animals has been documented for dispersing red wolves (*Canis rufus*;^14^) and lions (*Panthera leo*;^15^), which avoided areas near roads and with higher human population densities less strongly than residents. Non-resident gray wolves (*C. lupus*) similarly displayed reduced selection against human disturbance compared to resident wolves^16,17^. Alternatively, dispersers may not avoid areas of higher human disturbance at all; resident brown bears (*Ursus arctos*) avoided public roads and resident Iberian lynx (*Lynx pardinus*) avoided low-traffic roads, while dispersers did not^18,19^.

Gray wolves historically occupied the Northern Hemisphere north of 11–20° N, though by 1970 wolves were extirpated from most of their historical range in the contiguous USA^20^. Following federal protection in 1974, wolves recolonized additional areas of Minnesota, and former range in Wisconsin^21^ and the Upper Peninsula of Michigan^22^. The western Great Lakes population appears to have stabilized at around 4200 wolves^23^. Unoccupied habitat within former wolf range has been identified in the eastern USA, with apparent limited connectivity to current wolf range in the Great Lakes region^24^. However, estimates of habitat availability and connectivity should further consider factors underlying habitat selection including potential differences between resident and non-resident movements.

Gray wolves are territorial, though most disperse from their natal territory and establish or become residents of different territories^25^. Other wolves do not establish new territories or join existing territories and exhibit nomadic (or floating) movements, constrained by conspecific territories^20,26^. Wolves also make extraterritorial excursions (i.e., predispersal movements) of varying distance and duration^27,28^. Greater use of human-disturbed areas by wolves when dispersing or floating could result from avoiding existing wolf territories in less human-disturbed areas or decreased site familiarity that reduces their ability to avoid human disturbances, compared to when they are resident of a territory^8,29^. Alternatively, disturbances such as roads may facilitate efficient travel for non-residents^30^, while areas with high livestock abundance may provide food when lower site familiarity or prey abundance limits acquisition of wild prey^29^.

We investigated habitat use by gray wolves in the western Great Lakes region exhibiting resident (territorial) or non-resident (dispersing or floating) movements relative to human disturbance. We predicted wolves would select for areas with greater natural land cover and against areas of greater human disturbance as indexed by road densities and proportions of agricultural land cover, with stronger selection during resident than non-resident movements. We also quantified habitat selection and connectivity throughout the western Great Lakes region and evaluated these results against an existing connectivity map for wolves in the eastern USA. We expected a strong correlation between a previous connectivity map developed using winter track surveys^24^ and one resulting from this habitat selection analysis based on telemetry data.

## Methods

### Study area

**Figure 1:**
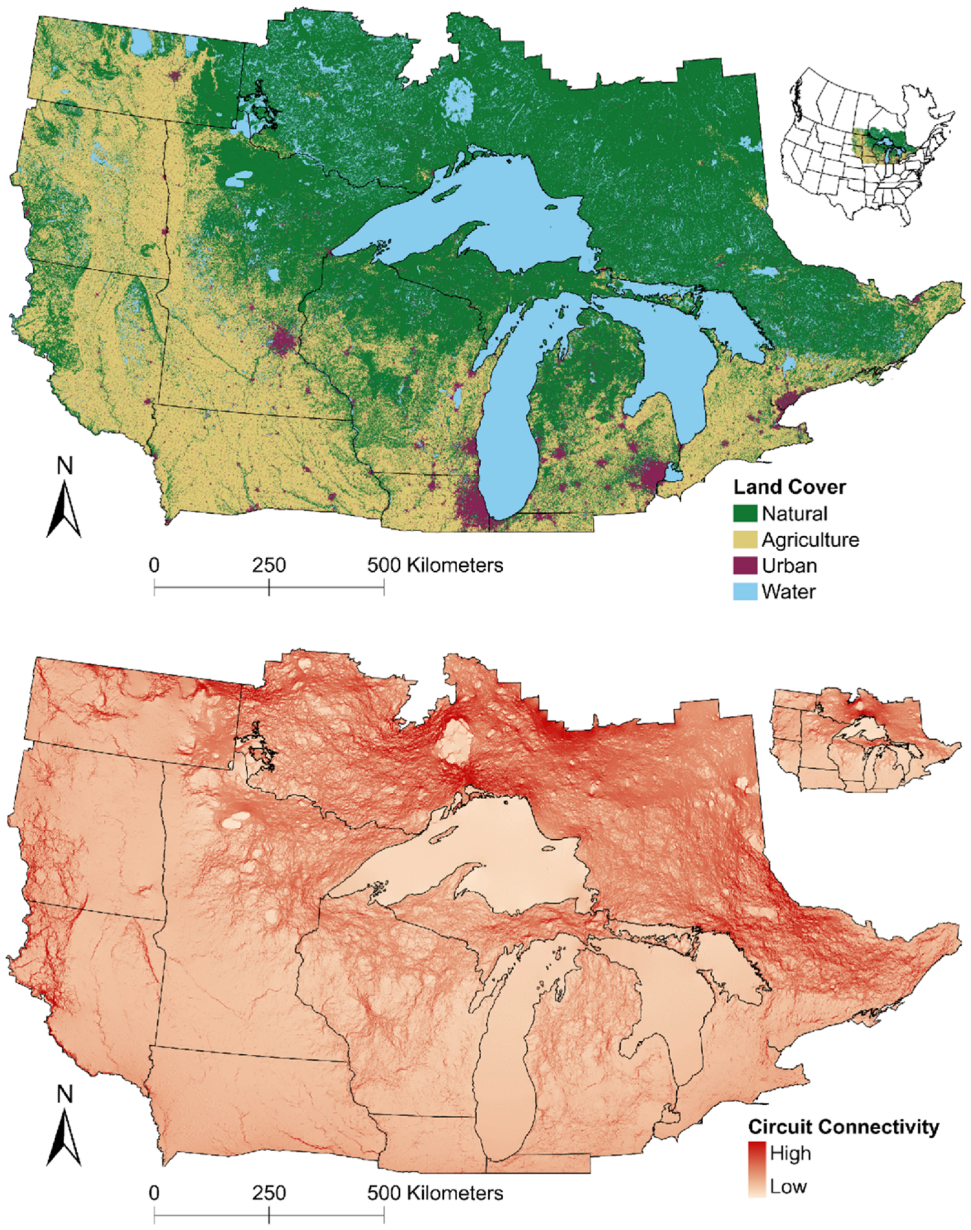
Top panel: land cover within the western Great Lakes distinct population segment of gray wolves (*Canis lupus*), USA and southern Ontario and Manitoba, Canada. Bottom panel: Circuit connectivity for the western Great Lakes distinct population segment of gray wolves (*Canis lupus*), USA and southern Ontario and Manitoba, Canada, 2017–2021. Figure based on a step-selection function (main figure) and circuit connectivity map derived from the same study area based on snow track data (^24^; inset). Figures were created using ArcGIS Pro 3.0.0 (https://www.esri.com).

The study area (Fig. 1) included the area representing the western Great Lakes distinct population segment of gray wolves (hereafter, western Great Lakes region;^31^), including Minnesota (220,185 km^2^), Wisconsin (145,593 km^2^), and Michigan (151,279 km^2^), and parts of North Dakota (108,193 km^2^), South Dakota (93,571 km^2^), Iowa (99,971 km^2^), and Illinois (27,190 km^2^).

The study area also included southern Ontario, Canada (515,966 km^2^), delineated by the Area of the Undertaking (the area in Ontario under forest management;^32^), and southern Manitoba (84,920 km^2^). The climate is predominantly humid continental, with warm summers and cold winters^33^. Average summer (June–September) minima are 7–17 ° C and maxima are 17–30 °C while average winter (December–March) minima are – 25 to − 6 °C and maxima are – 10 to 4 °C^34^. Elevations are 30–757 m above sea level^35^. The study area, excluding the Great Lakes, contains 46% natural land cover (primarily various forest types and wetlands) and 18% water, while agricultural and urban areas comprise 32% and 4%, respectively^36^. The primary prey of wolves in the study area is white-tailed deer (*Odocoileus virginianus*), in addition to American beaver (*Castor canadensis*), moose (*Alces alces*, where available), and other mammals^37^. Average wolf mid-winter pack size across Minnesota, Wisconsin, and Michigan is 2.7–5.6 individuals^38–40^, and the combined population is about 4200 wolves^23^.

### Data collection and processing

We used gray wolf GPS telemetry data collected during 2017–2021 by state, federal, and tribal agencies of Minnesota, Wisconsin, and Michigan. Animal capture and handling for data collection during original research and monitoring were approved by the respective state, tribal, and federal agencies. Use of these data for this study was approved by the Michigan State University Institutional Animal Care and Use Committee. We excluded the first five days of post-capture data from each wolf to reduce potential capture effects^3^. We created a dataset of 96 wolves (51 males, 39 females, 6 unknown) collared in Michigan (44), Wisconsin (31), and Minnesota (21), with 13- or 16-h relocation intervals. We compared two preliminary models using datasets with 13- or 16-h relocation intervals, found no notable differences, and pooled these datasets for analysis.

We separated resident (territorial) from non-resident (dispersing or floating) annual wolf movements by calculating relative net squared displacement (rNSD), which represents the squared Euclidian distance between consecutive locations^41^. For each wolf during each biological year (starting 15 April), we used the MigrateR package^42^ in program R^43^ to fit data to three a priori non-linear models representing resident, dispersing, and floating (named ‘nomadic’ in the MigrateR package) movements^41^. The rNSD assigns data to the model with the most similar net-squared displacement curve. For example, a curve whereby rNSD values are low and stable, exponentially increase, and thereafter stabilize at high rNSD values is classified as a disperser, as this is a curve typical of an animal that was resident to a territory, then dispersed, and thereafter settled into a new territory with a high relative distance from the first location in the dataset. After fitting data to each of the a priori curves, the best supported movement type is assigned based on the lowest AIC score^44^.

When data could not be assigned to a movement type because wolves displayed multiple movement types within a biological year, we split data between multiple movement categories based on visual inspection of the rNSD-plots and raw GPS data^42^. Visual inspection of movement data to confirm rNSD classifications is recommended to override rNSD classifications when suspected to be incorrect^42^. We then calculated 90% bivariate normal kernel utilization distributions to approximate annual range size using the ‘kernelUD’ function in AdehabitatHR package^45^. State reports during 2017–2021 were used as an independent source to set the maximum annual range size for wolves to be considered resident: from these reports we extracted the maximum territory size (561 km^2^) found across Minnesota, Wisconsin, and Michigan^46^. We classified movements within annual ranges ≤ 561 km^2^ as resident and reclassified movements within larger annual ranges initially classified as resident movements as floating movements. We classified extraterritorial movements between a territory and a non-overlapping territory as dispersal movements, from the first movement beyond the initial territory to the last movement before entering the subsequent territory. We included potential predispersal movements^27,28^ as dispersal movements by including extraterritorial movements leaving from and returning to the same territory, with a duration ≥ 10 days, based on visual inspection.

### Modeling landscape use

We used step-selection functions (SSF), linking consecutive animal locations and contrasting each observed step with three random available steps^47^. To obtain random steps, we pooled individual movements by movement type^47^ then randomly sampled the length and angle of random steps from the distribution of observed steps for each type. We used road density, proportion of natural land cover, and proportion of agricultural cover as continuous variables, whereby proportional land covers were calculated as the percentage of respective land cover types within a cell. We achieved this by assigning the value ‘1’ to the land cover of interest and ‘0’ to other land covers at the original raster resolution (30-m), after which we summed all values in an aggregated raster with 300-m resolution. We used road data from TIGER/line shapefiles (50-m resolution;^48^) and the Canadian National Road Network (5-m resolution;^49^), the most comprehensive road databases for these countries including categories ranging from highways to service roads, and roads only accessible by four-wheel drive vehicles. We used the North American land change monitoring system (NALCMS; 30-m resolution)^36^ to calculate proportional land cover. We reclassified land covers as natural (managed and unmanaged ‘forest’ classes, ‘shrubland’, ‘grassland’, ‘barren land’, and ‘wetland’), agricultural (class ‘cropland’), urban, and water (Fig. 1). We resampled rasters to 300-m resolution to reduce spatial mismatch between species and environmental data^50^, and rescaled continuous variables (− 1 to 1) to facilitate effects comparisons.

We fit the SSF using a conditional Poisson regression model, which yields equivalent estimates to the conditional logistic regression model typically used for SSFs^51^. We included random slopes for the continuous variables^51^ to account for individual variation among wolves. We fit the model using the glmmTMB package^52^ in program R. We used variance inflation factors (VIF) and pairwise correlations to test for multicollinearity of variables with thresholds of 10 and 0.70, respectively^53^. We selected from two candidate models based on the lowest AIC, or the competing model (ΔAIC < 2) with fewer terms^44^: one model contained road density and proportion of natural land cover, and another model contained these variables interacting with movement type. We created used-habitat calibration plots (UHC) to visualize how well model predictions characterize used locations by plotting the distribution of an explanatory variable at used locations and overlaying this with the distribution of explanatory variable values predicted by the model^54^. As a measure of ecological importance of statistical estimates, we spatially predicted relative strength of selection (RSS;^55^) throughout the study area by calculating RSS as the probability of selecting a given point over a point with average variable values in our study area, scaling probabilities from 0 to 1.

### Modeling connectivity

We used Circuitscape software to assess landscape-level connectivity without the assumption of animals having landscape knowledge^56^. We inverted the RSS surface raster to obtain an estimate of movement resistance^57^ then replaced each cell from the movement resistance surface with nodes connected by resistors, translating connectivity to ‘current flow’. We limited connectivity analysis to non-resident movements if the results of our SSF indicated significant differences (α < 0.05) in habitat selection for resident and non-resident wolf movements. We incorporated part of Indiana (8795 km^2^) into the study area to avoid a spatial interruption that would bias the circuit connectivity model. We assigned 154 points at about 40-km intervals along the perimeter of the study area and calculated connectivity between all pairs of points, providing an omnidirectional connectivity map for animals moving randomly through the landscape^56^. Connectivity between perimeter points is prone to edge effects as connectivity increases near these points. We therefore placed perimeter points at the midpoint of a 15-km buffer bordering the study area edge, filled cells within this buffer with the average movement resistance value of the study area, and removed this buffer after analysis^57^. To assess how the connectivity map compared to a previous species distribution model (SDM) and circuit connectivity analysis for gray wolves in the eastern USA and southern Canada^24^, we resampled our RSS and circuit connectivity maps to 1-km resolution and calculated Pearson’s correlation coefficients between the RSS and SDM rasters and their respective circuit connectivity rasters.

### Results

We retained 24,540 steps, with a median of 878 steps per wolf (range = 156–3811 steps). Of these steps, 16,668 were classified as resident, 1656 as dispersing, and 6216 as floating movements, with median step-lengths of 609, 1011, and 655 m for resident, dispersing, and floating movements, respectively. Resident, dispersing, and floating movements were found for 72, 20, and 24 wolves, respectively, and 17 of 96 wolves displayed multiple movement types. We used a maximum annual range size of 561 km^2^ for resident wolves to support movement type classification using the NSD-method and visual inspection, though average estimated annual range sizes were smaller for wolves classified as resident (mean = 195 km^2^, StDev = 116) than for dispersers (9294 km^2^, StDev = 15,065) or floaters (8608 km^2^, StDev = 11,119). Short-distance dispersal events occurred (n = 16, median = 54 km), with the longest dispersal being 615 km. Proportions of natural (VIF = 3.36) and agricultural (VIF = 3.03) cover were correlated (*r* = − 0.80), and we retained proportion of natural cover as it is inverse to the combined proportions of agricultural and urban cover, thus a stronger proxy of human disturbance.

**Figure 2:**
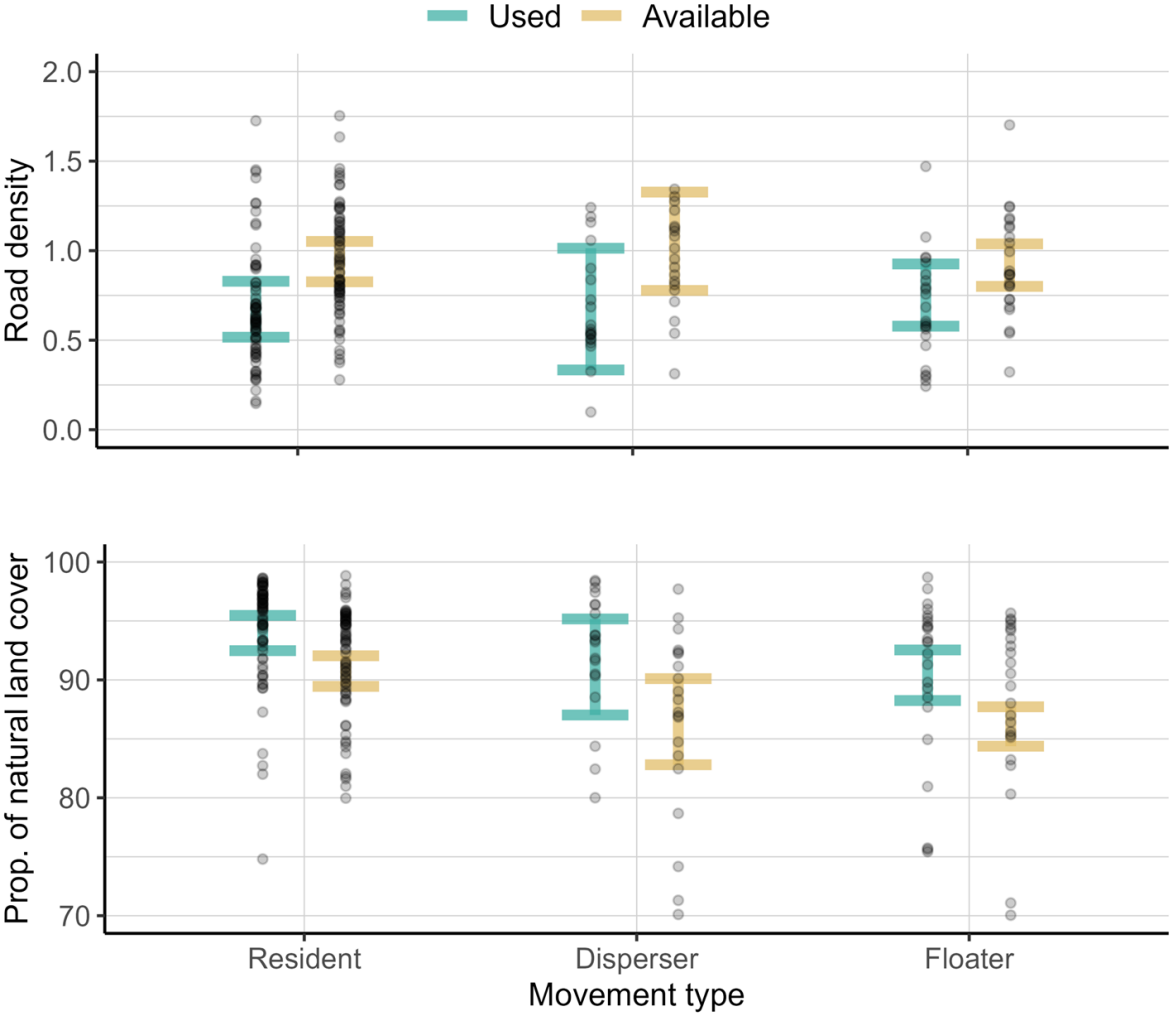
Characteristics of used and available steps for road density (km/km^2^, top panel) and proportion of natural cover (0–100, bottom panel) for 96 Gy wolves (*Canis lupus*) in the western Great Lakes region, USA, and southern Ontario and Manitoba, Canada, 2017–2021. Circles represent average selection of wolves by movement type, and error bars are 95% confidence intervals of these averages.

**Figure 3:**
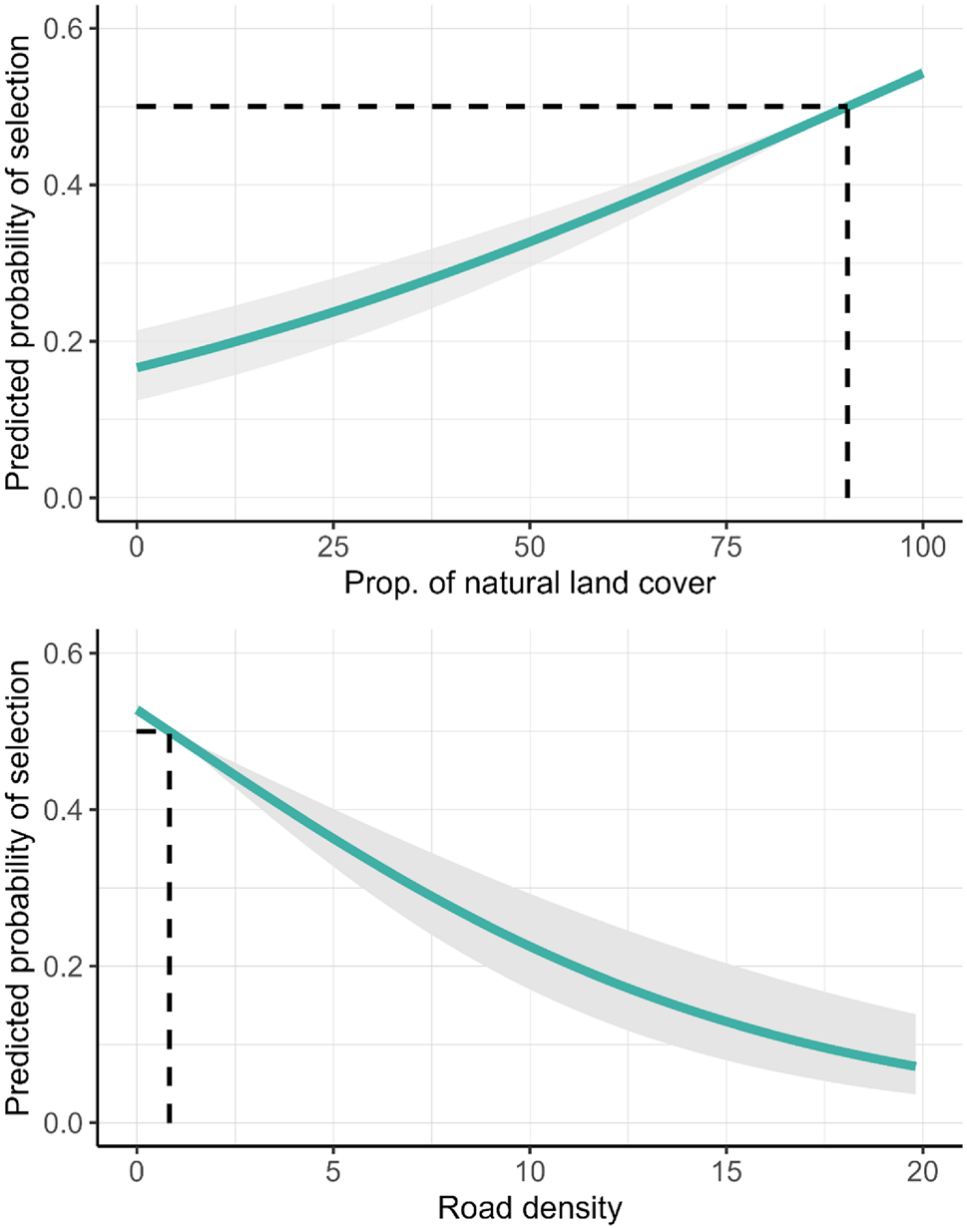
Predictions from a step-selection function for gray wolves (*Canis lupus*) in the western Great Lakes region, USA, and southern Ontario and Manitoba, Canada, 2017–2021. Predicted values are probabilities of selection relative to the average variable value of used and available steps (dashed lines, average proportion of natural land cover = 90.42%, average road density = 0.83 km/km^2^).

The model retaining movement type interacting with proportion of natural land cover and road density indicated no habitat selection differences among resident, dispersing, and floating wolves (Table 1; Fig. 2). Our final model included road density and proportion of natural land cover without interactions with movement type, and had a lower AIC value than the model including these interactions (ΔAIC = 5.2). This final model suggested wolves avoided areas with greater road density and selected for areas with greater proportions of natural cover (Fig. 3).

**Table 1:** Model selection results comparing used and available steps within the western Great Lakes distinct population segment of gray wolves (*Canis lupus*), USA and southern Ontario and Manitoba, Canada, 2017–2021.

Top model AIC = 454,149.5			
Parameter	Estimate	SE	P-value
Prop. natural cover	0.335	0.033	< 0.001
Road density	− 0.175	0.025	< 0.001

Second model ΔAIC = + 5.2			
Parameter	Estimate	SE	P-value
Prop. natural cover	0.335	0.033	< 0.001
Road density	− 0.175	0.025	< 0.001
Road density × Floating	0.031	0.052	0.557
Prop. natural cover × Floating	− 0.069	0.064	0.275
Road density × Dispersing	0.037	0.061	0.548
Prop. natural cover × Dispersing	0.017	0.050	0.734

Models were ranked using AIC; variables included road density (km/km^2^) and proportion of natural cover, and their interactions with movement type (reference level: Resident). Continuous variables were scaled (− 1 to 1) and included random slopes for continuous variables to account for individual variation among wolves. Parameter estimates are reported with standard error (SE) and p-values (α < 0.05).

Calibration of our top-ranked model was successful for road density and reasonable for proportions of natural cover, based on visual inspection of overlap between predicted and used values in UHC plots (Appendix A).

Areas previously estimated suitable that were not selected by wolves included parts of the Lower Peninsula of Michigan, and isolated and fragmented areas east of the Missouri river in North and South Dakota. Non-resident movements in our dataset were limited to established wolf range. Because we detected no differences in habitat selection between resident and non-resident movements, we retained all data for connectivity analysis. Our circuit connectivity map indicated highest connectivity for wolves in the northern and eastern parts of Ontario and was positively correlated (*r* = 0.75) with the circuit connectivity raster of the SDM (Fig. 1). The resampled RSS raster (Appendix B) was positively correlated (*r* = 0.78) with a previous habitat suitability raster^24^.

## Discussion

Our estimate of gray wolf habitat selection in the western Great Lakes region of the USA and southern Ontario and Manitoba, Canada, supported our prediction that wolves avoid areas with high road densities and select for areas with high proportions of natural cover. Contrary to our prediction, we found no differences among habitat selection of wolves exhibiting resident, dispersing, or floating movements. We also confirmed unoccupied habitat in the western Great Lakes region is limited, and connectivity between occupied and unoccupied habitat is constrained by the Great Lakes, areas of extensive agriculture, and urban areas.

Habitat selection of resident and non-resident wolves in our study was similar, though previous studies found it can differ by disturbance type. Gray wolves in Portugal displayed increased tolerance toward roads and settlements during dispersal, but not towards areas with higher livestock densities or windfarms^11^. The absence of increased tolerance for high road densities or proportions of urban or agricultural land cover during non-residency may be due to non-resident movements in our study occurring within established wolf range, causing high similarity or overlap in habitat characteristics to which residents and non-residents are exposed. Only four dispersing wolves exceeded estimated mean dispersal distances (range = 29–148 km) for the western Great Lakes region^58^. The prevalence of short-distance dispersals (median = 54 km), and dispersal and floating movements being largely limited to established wolf range, may be caused by increased opportunities for non-residents to join existing packs due to high pack densities^59,60^. Alternatively, with little unoccupied habitat available, non-residents may traverse conspecific territories when higher prey availability or lower human disturbance in occupied habitat outweighs risks of encountering conspecifics^17^.

The relative selection strength (RSS) map was positively correlated with a habitat suitability map for the same area based on wolf winter track surveys^24^, while derived connectivity maps were also positively correlated. This similarity provides validation of current wolf range and habitat connectivity throughout the western Great Lakes region from independent data. Our results also suggest that accounting for differences between resident and non-resident movements using data transformations^61^, or by limiting connectivity analyses to non-resident movement data^15,16^, may be unnecessary when non-resident movements are largely limited to established range or otherwise similar to resident individuals.

We note several limitations to our study. Similar to how movement resistance rasters are calculated from habitat suitability maps^24^, we calculated a resistance raster by inverting the RSS map resulting from our SSF^62^. This is not ideal because RSS values are conditional probabilities^55^, though no alternative approach is available. Additionally, we used a traditional step-selection function that builds a habitat selection analysis upon estimation of movement, which can introduce a bias in habitat selection because habitat selection to an extent may depend on the movement capability of animals^63^. A fully mechanistic approach to classifying movement types is unavailable, and the migrateR package was developed primarily for migratory animals, so classification depends in part on visual interpretation of data^42^. Also, wolves can swim up to 2 km^64^ but can cross larger waterbodies during freeze-over^65^. Our approach resulted in waterbodies having above average resistance to movement due to low natural, terrestrial cover, but as the Great Lakes are roadless they have a lower resistance than areas with low natural cover and high road density. Year-round estimates of connectivity are imperfect due to seasonal changes in movement resistance of water. Using GPS locations collected at shorter intervals could be used to assess finer-scale wolf movements, and may reveal differences in habitat selection among movement types^11^. Analysis including non-resident movements beyond established range also is needed to confirm whether differences in habitat use between resident and non-resident wolves depend on differences in the range of conditions they occur in. Finally, wolves generally avoid higher road densities but can select for minor, lower traffic roads for efficient travel^66^. The road databases used here generally group unpaved rural roads, that wolves are known to use, with roads in suburban areas that wolves would likely avoid, thus testing the response to road densities classified by road type was not possible.

We suggest potential for further recolonization of the western Great Lakes region is low, as unoccupied habitat and habitat connectivity are limited. The Straits of Mackinac can connect current range in the Upper Peninsula of Michigan with the Lower Peninsula during freeze-over, though recent crossings of the straits have been too infrequent for population establishment^67^. Recolonization of potential habitat in North and South Dakota is limited by low dispersal frequencies and high anthropogenic mortality^68^, and connectivity with current range may be higher through Manitoba than through Minnesota.

## Conclusions

We offer further support that gray wolves in the western Great Lakes region select for areas with high proportions of natural cover, and against human disturbance as indexed by road densities. We found no differences in habitat selection among wolves that were resident, dispersing, or floating. The need to limit connectivity analyses to non-resident movements, or to apply transformations to data of primarily resident wolf movements, will depend on the magnitude of differences in habitat characteristics experienced by resident and non-resident individuals. As most wolf habitat in the western Great Lakes region appears occupied and there is limited habitat connectivity between currently occupied range and limited unoccupied range in the USA part of the western Great Lakes region, further recolonization appears most likely through Canada to connect with wolf habitat in North Dakota, and across the Straits of Mackinaw to connect with habitat in the Lower Peninsula of Michigan. Interjurisdictional cooperation will be important to improve landscape connectivity for gray wolves between Canada and the USA. If recolonization of areas beyond current wolf range in the Great Lakes region is desired, promoting human-wolf co-existence in areas most likely to be recolonized is pertinent, though further natural recolonization within and beyond the Great Lakes region appears limited by the dominance of urban and agricultural areas surrounding current range.

### Supplementary Information

Supplementary Information.

## Data Availability

Data supporting the conclusions of this article are not publicly available as the subject species (gray wolf) is federally protected under the Endangered Species Act, and subject to poaching within the study area. Data are available upon request from co-authors affiliated with the Department of Natural Resources of Minnesota (john.erb@state.mn.us), Wisconsin (david.macfarland@wisconsin.gov), and Michigan (petroeljet@michigan.gov).
