# Supplementary material for: Habitat selection of resident and non-resident gray wolves: implications for habitat connectivity
Source: Sci Rep. 2023 Nov 21;13:20415. doi: 10.1038/s41598-023-47815-0 (PMC10663587; doi:10.1038/s41598-023-47815-0)

Supplemental information

Manuscript title:

Habitat selection of resident and non-resident gray wolves: implications for habitat connectivity

Appendix A. Used-habitat calibration plots for proportion of natural cover (top panel) and road density (km/km^2, bottom panel) used in a step-selection function for gray wolves (*Canis lupus*) in the western Great Lakes region, USA, and southern Ontario and Manitoba, Canada, 2017–2021.

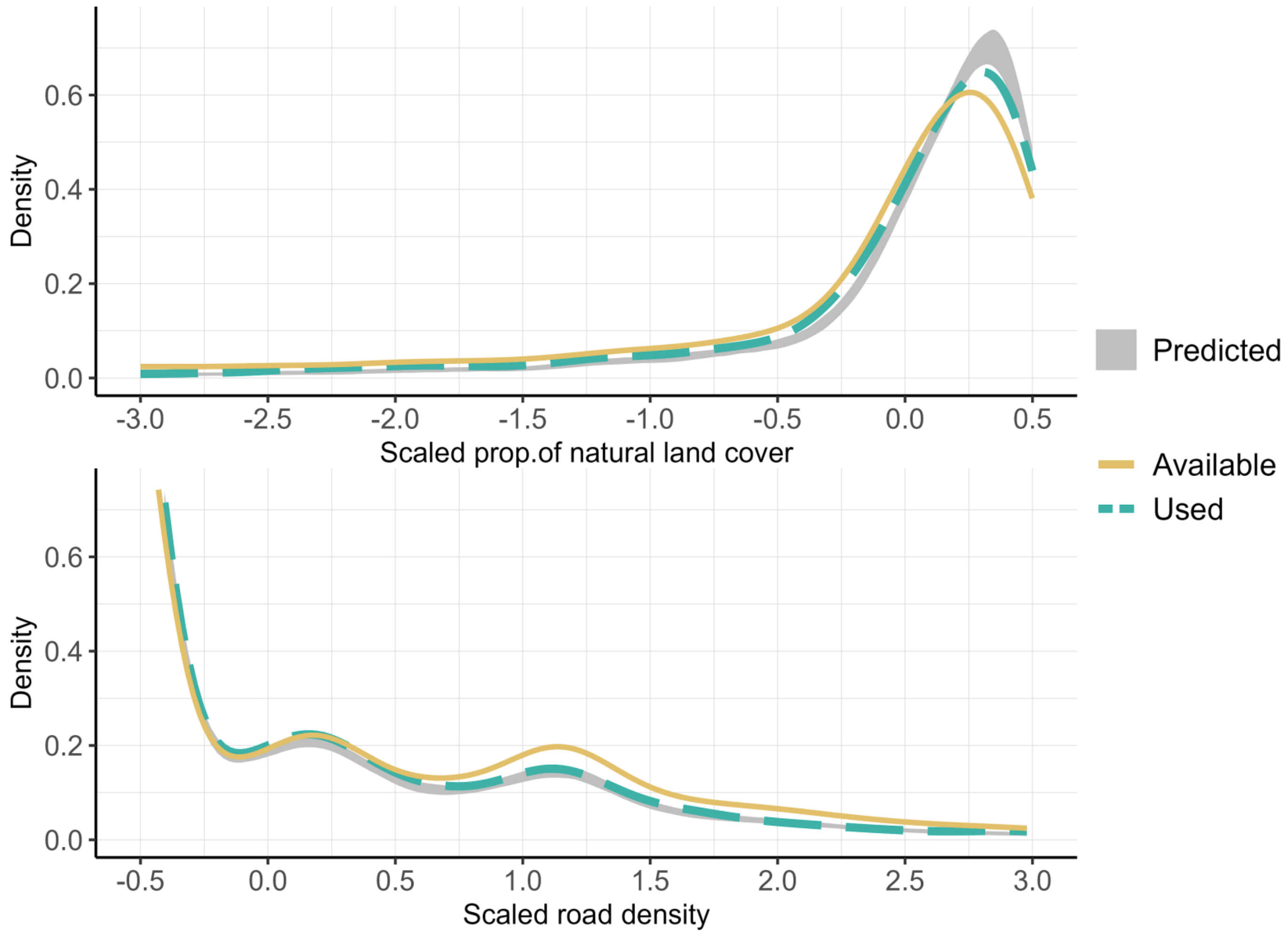

Appendix B. Predicted relative selection strength (RSS) for the western Great Lakes distinct population segment of gray wolves (*Canis lupus*), USA and southern Ontario and Manitoba, Canada based on a step-selection function, along with an approximate southern border of wolf range. Figures were created using ArcGIS Pro 3.0.0 (https://www.esri.com).

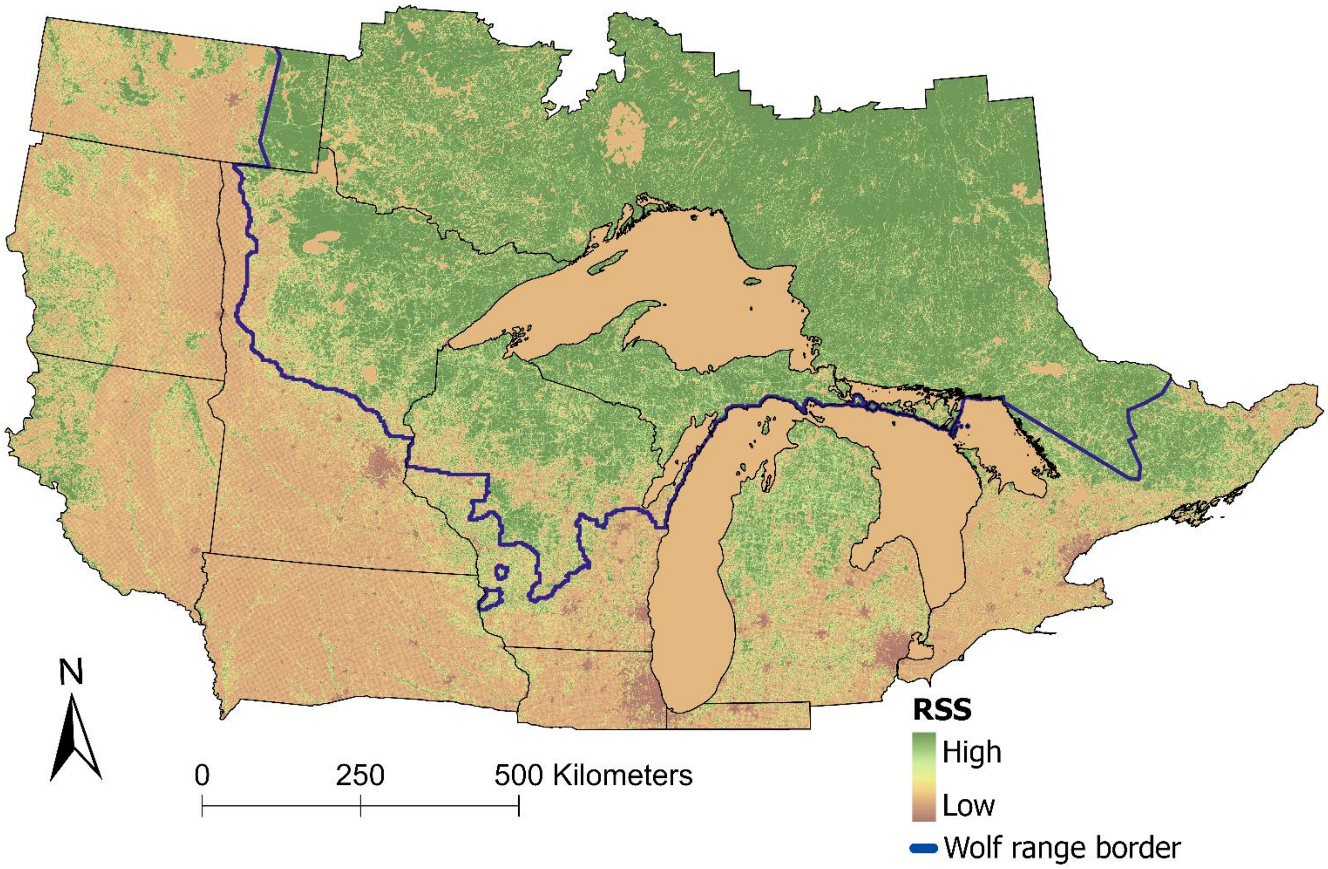

Supplement: Supplementary file 1 — Supplementary Information. [file 41598_2023_47815_MOESM1_ESM.pdf]
